# Hemoglobin A1c and preoperative glycemia as a decision tool to help minimise sternal wound complications: a retrospective study in OPCAB patients

**DOI:** 10.1186/s13019-021-01580-1

**Published:** 2021-07-20

**Authors:** Jef Van den Eynde, Abel Van Vlasselaer, Annoushka Laenen, Delphine Szecel, Bart Meuris, Tom Verbelen, Steven Jacobs, Peter Verbrugghe, Wouter Oosterlinck

**Affiliations:** 1 Department of Cardiovascular Diseases, Research Unit of Cardiac Surgery, University Hospitals Leuven, Herestraat 49, 3000 Leuven, Belgium; 2 Leuven Biostatistics and Statistical Bioinformatics Centre (L-BioStat), KU Leuven, Leuven, Belgium

**Keywords:** Diabetes mellitus, Glycemia, HbA1c, OPCAB, Sternal wound complications, Sternal wound infections

## Abstract

**Background:**

Poor glycemic control has been associated with an increased risk of wound complications after various types of operations. However, it remains unclear how hemoglobin A1c (HbA1c) and preoperative glycemia can be used in clinical decision-making to prevent sternal wound complications (SWC) following off-pump coronary artery bypass grafting (OPCAB).

**Methods:**

We conducted a retrospective study of 1774 consecutive patients who underwent OPCAB surgery between January 2010 and November 2016. A new four-grade classification for SWC was used. The associations of HbA1c and preoperative glycemia with incidence and grade of SWC were analysed using logistic regression analysis and proportional odds models, respectively.

**Results:**

During a median follow-up of 326 days (interquartile range (IQR) 21–1261 days), SWC occurred in 133/1316 (10%) of non-diabetes and 82/458 (18%) of diabetes patients (*p* < 0.001). Higher HbA1c was significantly associated with a higher incidence of SWC (odds ratio, OR 1.24 per 1% increase, 95% confidence interval, CI 1.04;1.48, *p* = 0.016) as well as a higher grade of SWC (OR 1.25, 95% CI 1.06;1.48, *p* = 0.010). There was no association between glycemia and incidence (*p* = 0.539) nor grade (*p* = 0.607) of SWC. Significant modifiers of these effects were found: HbA1c was associated with SWC in diabetes patients younger than 70 years (OR 1.41, 95% CI 1.17;1.71, *p* < 0.001), whereas it was not in those older than 70 years. Glycemia was associated with SWC in patients who underwent non-urgent surgery (OR 2.48, 95% CI 1.26;4.88, *p* = 0.009), in diabetes patients who received skeletonised grafts (OR 4.83, 95% CI 1.28;18.17, *p* = 0.020), and in diabetes patients with a BMI < 30 (OR 2.19, 95% CI 1.01;4.76, *p* = 0.047), whereas it was not in the counterparts of these groups.

**Conclusions:**

Under certain conditions, HbA1c and glycemia are associated SWC following OPCAB. These findings are helpful in planning the procedure with minimal risk of SWC.

## Background

Sternal wound complications (SWC) remain a feared complication after coronary artery bypass grafting (CABG). According to the extensiveness of the infection, SWC can be classified into superficial, deep, and organ/space infections [1]. Deep incisional infection and mediastinitis are the least common and are usually reported together as deep SWC (DSWI) [2]. Although the incidence of DSWI is only 0.5–3%, it is associated with a significant risk of mortality, prolonged hospital stay, and increased medical costs [3–5]. In fact, any type of SWC can be a considerable burden for the patient.

The process of wound healing is complicated and requires the integration of various overlapping events [6]. Although several checks and balances are present, the process can be disrupted by conditions such as vascular insufficiency, immunodeficiency, stress, and chronic diseases. Especially type 2 diabetes mellitus has become an increasingly common risk factor in the population undergoing CABG, as currently one out of two CABG patients have diabetes [7]. The glycemic fluctuations and prolonged hyperglycemia which are typical for diabetes can trigger oxidative stress and promote pro-inflammatory responses [8]. The idea that glycemic dysregulation can increase the risk of poor wound healing is further supported by many studies that have associated diabetes with increased rates of SWC following CABG [9–11].

Poor glycemic control results in an elevated hemoglobin A1c (HbA1c), a biomarker which is widely used to diagnose and follow up on the treatment of diabetes patients [12]. As glycemia and HbA1c are markers of acute and chronic glycemic control, respectively, it has been hypothesized that they might be useful tools in clinical decision making regarding the prevention of SWC. However, it remains unclear how HbA1c and preoperative glycemia can be used in clinical decision-making to prevent SWC. The aim of this study was to investigate the association of HbA1c and preoperative glycemia in a population undergoing off-pump coronary artery bypass grafting (OPCAB).

## Methods

### Population and study design

This study conforms to the ethical guidelines of the 1975 Declaration of Helsinki as reflected in a priori approval by the local Ethical Committee of the University Hospitals of Leuven. The data from 1900 consecutive patients who underwent OPCAB surgery at the University Hospitals of Leuven between January 2010 and November 2016, were studied. All diabetes and non-diabetes patients who had received one or more conduits were eligible for inclusion. We excluded 126 patients who did not have at least one glycemia or HbA1c value, thus leaving 1774 patients in our database: 1316 non-diabetes patients and 458 diabetes patients. Data on patient follow-up was obtained through available information in their electronic medical records, as well as hospitalisations and outpatient surgical and cardiology consultations. Minimal period of follow-up for every patients was until hospital discharge or death.

Demographic characteristics and perioperative variables were considered and compared between both groups. Demographics included age, gender and body mass index (BMI). Comorbidities such as diabetes, organ transplantation, chronic obstructive pulmonary disease (COPD) with need of bronchodilator treatment, and use of oral corticosteroids were analysed. Perioperative information contained the harvesting method, the use of single (SIMA) or bilateral internal mammary arteries (BIMA), and whether the surgery was an urgency. The major postoperative outcomes were the incidence and grade of SWC. Another outcome was the overall survival.

The primary variables of interest were preoperative glycemia and HbA1c. All selected HbA1c values had been measured within a time frame of 3 months from the date of surgery. In the case of multiple available HbA1c values, the value closest to the date of surgery was selected. HbA1c was also collected in a subset of non-diabetes patients. In the analyses, values > 300 mg/dL for glycemia were regarded as outliers and excluded.

Because commonly used classifications such as the STS classification of deep sternal wound infections only include severe types of infection, we have previously introduced a new classification for a more comprehensive definition of wound complications [13]. This classification, as given in Table 1, consists of four severity grades. Grade 1 and 2 are mild and superficial sternal wound problems or infections with minimal impact on patient recovery, while grade 3 and 4 are severe and deep complications that need subsequent surgical interventions. The majority of the SWC were diagnosed at the time of hospitalisation.

**Table 1:** Grading of sternal wound complications

Grade 1: Minor	Superficial wound problem: local redness or minimal drainage
	Conservative approach, spontaneous healing
Grade 2: Superficial	Wound infection: positive culture
	Antibiotic treatment
Grade 3: Moderate	Deep wound infection: dehiscence
	Need for drainage, debridement, or VAC
Grade 4: Severe	Mediastinitis or mechanical sternal dehiscence
	Refixation of the sternum or omentoplasty

*VAC* vacuum assisted closure

### Surgical procedure

All operations were led by one of 7 in-house cardiothoracic surgeons. As mentioned earlier, all patients underwent OPCAB surgery in which median sternotomy was the standard access strategy. The IMA was used as the main graft vessel for myocardial revascularisation. Either SIMA or BIMA were used. Between January 2010 and December 2014, conventional non-skeletonised technique was used to harvest the IMA in all patients (*n* = 1487). From January 2015 to November 2016, all patients (*n* = 413) received a skeletonised IMA graft. When the skeletonised technique was used, the IMA was isolated from the thoracic wall using meticulous dissection, leaving the adjacent vein, fat tissue, endothoracic fascia, parietal pleura and intercostal muscle undisturbed. Dissection was performed using electrocautery, and arterial side branches were divided using hemoclips and microscissors. In the non-skeletonised technique, the IMA was dissected along with the surrounding tissues using electrocautery. All IMA grafts were mobilised from the first rib to the bifurcation of the IMA into the superior epigastric and musculophrenic arteries.

Optimal wound management was pursued in all patients. Preoperative infection prophylaxis has widely remained the same in the time period this study covers. Patients had a preoperative chlorhexidine bath and the surgical site was clipped. Antibiotic prophylaxis was given by intravenous administration of 3 g of cefazolin before incision and 2 g every 3 h intraoperatively. Tight glycemic control was attained pre-, per- and postoperatively. Bone wax was used according to needs. The sternum was closed using 8 to 10 single steel wires, and subsequently the fascia and subcuticular tissues were approximated separately using a running suture.

### Statistical analyses

Continuous variables were checked for normality and the difference between groups was tested with the t-test or Mann-Whitney U test accordingly. Categorical variables are expressed as frequency and proportion, and differences were assessed with the Chi-square test. We analyzed SWC as an ordinal variable and as a dichotomous variable. The association between preoperative glycemia or HbA1c and the ordinal SWC grade was analysed using proportional odds models, whereas the incidence of SWC was investigated with logistic regression analysis. Adjustment was performed by using multivariable models, including these patient characteristics in the analysis model on which groups were different. Interactions of HbA1c and glycemia with other variables were also investigated. Analyses were performed for the entire study population, but also for non-diabetes and diabetes patients separately. All tests were two-sided and a *p*-value less than 0.05 was deemed statistically significant. All analyses have been performed using SAS software (version 9.4; SAS Institute, Cary, NC).

## Results

### Study population

Demographics of the study population are given in Table 2. Diabetes patients were comparable to non-diabetes patients for most variables except for a higher BMI (27.9 ± 4.93 kg/m^2^ vs 26.6 ± 4.04 kg/m^2^ for diabetes and non-diabetes, respectively, *p* < 0.001) and a lower use of BIMA grafts (54.8% vs 64.2%, *p* < 0.001). Furthermore, diabetes patients had a higher preoperative glycemia (131.3 ± 40.3 mg/dL vs 107.0 ± 24.3 mg/dL, *p* < 0.001) and HbA1c (6.91 ± 1.22% vs 5.80 ± 0.79%, *p <* 0.001).

**Table 2:** Demographic characteristics and perioperative variables

Variable	All (***n*** = 1774)	Non-diabetes (***n*** = 1316)	Diabetes (***n*** = 458)	***P***
**Age, years**	67.9 ± 9.65	68.0 ± 9.84	67.3 ± 9.09	0.068
**Male gender**	1433 (80.78)	1075 (81.69)	358 (78.17)	0.100
**BMI, kg/m**^**2**^	26.9 ± 4.33	26.6 ± 4.04	27.9 ± 4.93	< 0.001
**BMI group**				
< 25	486 (28.86)	390 (31.3)	96(21.9)	< 0.001
25–29	789 (46.85)	593 (47.6)	196 (44.7)	
30–34	331 (19.66)	220 (17.7)	111 (25.3)	
> 34	78 (4.63)	42 (3.37)	36 (8.20)	
**Urgent surgery**	864 (48.70)	630 (47.87)	234 (51.09)	0.235
**Oral corticosteroids**	145 (8.17)	103 (7.83)	42 (9.17)	0.366
**Transplantation**	16 (0.90)	8 (0.61)	8 (1.75)	0.026
**COPD**	110 (6.20)	78 (5.93)	32 (6.99)	0.418
**Skeletonisation**	384 (21.65)	271 (20.59)	113 (24.67)	0.068
**BIMA**	1096 (61.78)	845 (64.21)	251 (54.80)	< 0.001
**HbA1c (%)**	/	5.80 ± 0.79^a^	6.91 ± 1.22	< 0.001
**Glycemia (mg/dL)**	107.0 ± 24.3	107.0 ± 24.3	131.3 ± 40.3	< 0.001

*BMI* Body mass index, *COPD* chronic obstructive pulmonary disease, *BIMA* bilateral internal mammary artery bypass grafting

^a^data from 129 non-diabetes patients

Values are presented as mean ± SD or n (%)

*P* < 0.05 was considered significant

### Association of hemoglobin A1c and glycemia with sternal wound complications

In this cohort, the median follow-up was 326 days (interquartile range (IQR) 21–1261 days). During follow-up, SWC occurred in 215 (12.12%) patients: 133 (10%) of non-diabetes and 82 (18%) of diabetes patients (*p* < 0.001). Table 3 lists the incidence of SWC per grade.

**Table 3:** Outcomes: sternal wound complication incidence and grade

Variable	All (***n =*** 1774)	Non-diabetes (***n*** = 1316)	Diabetes (***n*** = 458)	***P***
**SWC incidence**	215 (12.12)	133 (10.11)	82 (17.90)	0.067
**SWC grade**				< 0.001
1	85 (4.79)	59 (4.48)	26 (5.68)	0.303
2	36 (2.03)	23 (1.75)	13 (2.84)	0.154
3	66 (3.72)	33 (2.51)	33 (7.21)	< 0.001
4	28 (1.58)	18 (1.37)	10 (2.18)	0.228

*SWC* sternal wound complication, *CI* confidence interval

Values are presented as mean ± SD or n (%) if not otherwise specified

*P* < 0.05 was considered significant

**Fig. 1:**
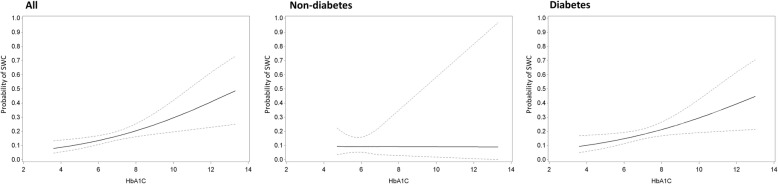
Estimated probability with 95% confidence interval for sternal wound complications as a function of preoperative HbA1c. The functions are given for all patients, the non-diabetes patients, and the diabetes patients

**Fig. 2:**
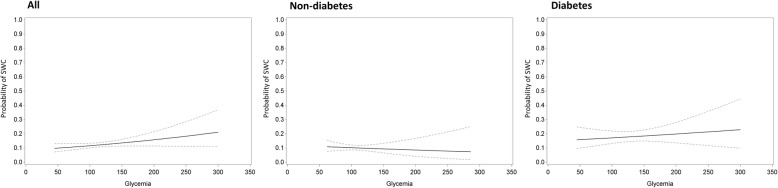
Estimated probability with 95% confidence interval for sternal wound complications as a function of preoperative glycemia. The functions are given for all patients, the non-diabetes patients, and the diabetes patients

Among the full study cohort, patients with SWC were more often female (27.0% vs 18.2%, for SWC and no SWC, respectively, *p* = 0.002), had a higher BMI (28.9 ± 5.03 kg/m^2^ vs 26.7 ± 4.15 kg/m^2^, *p* < 0.001), and had a higher use of BIMA grafts (75.4% vs 59.9%, *p* < 0.001) (Table 4). Those who developed SWC had a higher HbA1c (7.03 ± 1.42% vs 6.60 ± 1.18%, *p* = 0.007). The adjusted models revealed that a higher HbA1c was significantly associated with a higher incidence of SWC (odds ratio, OR 1.24 per 1% increase, 95% confidence interval, CI 1.04;1.48, *p* = 0.016) as well as a higher grade of SWC (OR 1.25, 95% CI 1.06;1.48, *p* = 0.010) (Table 5). Patients who developed SWC had similar preoperative glycemia (116.6 ± 35.5 mg/dL vs 112.8 ± 30.5 mg/dL, *p* = 0.094) (Table 4). The adjusted models revealed no association between preoperative glycemia and incidence (*p* = 0.539) nor grade (*p* = 0.607) of SWC (Table 5). The estimated probabilities with 95% confidence interval for sternal wound complications as a function of HbA1c and preoperative glycemia are represented in Figs. 1 and 2.

**Table 4:** Comparison between patients with and without sternal wound complications

Variable	All (***n =*** 1774)			Non-diabetes (***n =*** 1316)			Diabetes (***n =*** 458)		
	No SWC (***n =*** 1559)	SWC (***n*** = 215)	***P***	No SWC (***n*** = 1183)	SWC (***n*** = 133)	***P***	No SWC (***n*** = 376)	SWC (***n*** = 82)	***P***
**Age, years**	67.9 ± 9.62	67.1 ± 9.85	0.212	68.0 ± 9.81	68.2 ± 10.1	0.890	67.7 ± 9.03	65.4 ± 9.18	0.037
**Male gender**	1276 (81.9)	157 (73.0)	0.002	974 (82.3)	101 (75.9)	0.071	302 (80.3)	56 (68.3)	0.017
**BMI, kg/m**^**2**^	26.7 ± 4.15	28.9 ± 5.03	< 0.001	26.4 ± 3.93	28.0 ± 4.70	< 0.001	27.4 ± 4.70	30.3 ± 5.24	< 0.001
**BMI group**			< 0.001			< 0.001			< 0.001
< 25	439 (29.8)	47 (22.3)		355 (31.8)	35 (26.9)		84 (23.5)	12 (14.8)	
25–29	710 (48.2)	79 (37.4)		541 (48.5)	52 (40.0)		169 (47.2)	27 (33.3)	
30–34	277 (18.8)	54 (25.6)		189 (17.0)	31 (23.9)		88 (24.6)	23 (28.4)	
> 34	47 (3.2)	31 (14.7)		30 (2.7)	12 (9.2)		17 (4.8)	19 (23.5)	
**Urgent surgery**	756 (48.5)	108 (50.2)	0.632	562 (47.5)	68/133 (51.13)	0.428	194 (51.6)	40 (48.8)	0.644
**Oral corticosteroids**	123 (7.9)	22 (10.2)	0.240	88 (7.4)	15/133 (11.28)	0.118	35 (9.3)	7 (8.5)	0.826
**Transplantation**	12 (0.8)	4 (1.9)	0.113	7 (0.6)	1 (0.8)	0.822	5 (1.3)	3 (3.7)	0.145
**COPD**	95 (6.1)	15 (7.0)	0.615	67 (5.7)	11 (8.3)	0.227	28 (7.5)	4 (4.9)	0.408
**Skeletonisation**	346 (22.2)	38 (17.7)	0.131	244 (20.6)	27 (20.3)	0.930	102 (27.1)	11 (13.4)	0.009
**BIMA**	934 (59.9)	162 (75.4)	< 0.001	741 (62.6)	104 (78.2)	< 0.001	193 (51.3)	58 (70.7)	0.001
**HbA1c (%)**	6.60 ± 1.18	7.03 ± 1.42	0.007	5.80 ± 0.82^a^	5.80 ± 0.47^a^	0.757	6.85 ± 1.16^a^	7.21 ± 1.43^a^	0.081
**Glycemia (mg/dL)**	112.8 ± 30.5	116.6 ± 35.5	0.094	107.1 ± 24.6	106.1 ± 21.6	0.996	130.79 ± 39.1	133.8 ± 45.6	0.800

*BMI* Body mass index, *COPD* chronic obstructive pulmonary disease, *BIMA* bilateral internal mammary artery bypass grafting

^a^data from 129 non-diabetes patients

Values are presented as mean ± SD or n (%)

*P* < 0.05 was considered significant

**Table 5:** Association of hemoglobin A1c and glycemia with incidence and grade of sternal wound complications

Variable		All (***n*** = 1774)				Non-diabetes (***n*** = 1316)				Diabetes (***n*** = 458)			
		Incidence	***P***	Grade	***P***	Incidence	***P***	Grade	***P***	Incidence	***P***	Grade	***P***
**HbA1c (%)**^a^	Non-adjusted	1.28 (1.09;1.50)	0.003	1.29 (1.10;1.50)	0.002	1.00 (0.47;2.13)	0.992	1.01 (0.48;2.11)	0.979	1.25 (1.04;1.49)	0.018	1.249 (1.05;1.49)	0.014
	Adjusted^b^	1.24 (1.04;1.48)	0.016	1.25 (1.06;1.48)	0.010	0.85 (0.24;2.98)	0.794	0.91 (0.30;2.80)	0.875	1.22 (1.00;1.49)	0.054	1.23 (1.01;1.48)	0.035
**Glycemia (mg/dL)**	Non-adjusted	1.43 (0.94;2.16)	0.093	1.449 (0.96;2.19)	0.077	0.82 (0.37;1.82)	0.630	0.85 (0.39;1.86)	0.683	1.20 (0.67;2.14)	0.539	1.16 (0.65;2.07)	0.607
	Adjusted^b^	1.51 (0.95;2.42)	0.082	1.49 (0.94;2.37)	0.089	0.98 (0.39;2.50)	0.972	1.03 (0.41;2.58)	0.950	1.15 (0.60;2.19)	0.673	1.09 (0.58;2.05)	0.800

^a^data from 458 diabetes patients and 129 non-diabetes patients, for a total of 587 patients who had HbA1c values available

^b^adjusted for gender, BMI, and rate of BIMA use

*P* < 0.05 was considered significant

Within the group of diabetes patients, those who developed SWC were younger (65.4 ± 9.18 vs 67.7 ± 9.03 years, *p* = 0.037), were more often female (31.7% vs 19.7%, *p* = 0.017), had a higher BMI (30.3 ± 5.24 vs 27.4 ± 4.70, *p* < 0.001), underwent less frequently graft skeletonisation (13.4% vs 27.1%, *p* = 0.009), and the use of BIMA grafts was more often employed in this patient group (70.7% vs 51.3%, *p* = 0.001) (Table 4). There was a trend of higher HbA1c in those who developed SWC (7.21 ± 1.43% vs 6.85 ± 1.16%, *p* = 0.081). The unadjusted models revealed that higher HbA1c was significantly associated with a higher incidence of SWC (*p* = 0.018) as well as a higher grade of SWC (*p* = 0.014). However, the significance of this association disappeared in the adjusted model for incidence of SWC (OR 1.22, 95% CI 1.00;1.49, *p* = 0.054), whereas it remained for grade of SWC (OR 1.23, 95% CI 1.01;1.48, *p* = 0.035). Diabetes patients who developed SWC had similar preoperative glycemia (133.8 ± 45.6 mg/dL vs 130.8 ± 39.1 mg/dL). The models revealed no association between preoperative glycemia and incidence (*p* = 0.673) nor grade (*p* = 0.800) of SWC.

Within the group of non-diabetes patients, those who developed SWC had a higher BMI (28.0 ± 4.70 kg/m^2^ vs 26.4 ± 3.93 kg/m^2^) and a higher use of BIMA (78.2% vs 62.6%, *p* < 0.001). Preoperative glycemia was similar (106.1 ± 21.6 mg/dL vs 107.1 ± 24.6 mg/dL, *p* = 0.996). In addition, 129 of the non-diabetes patients had HbA1c, showing similar values in the SWC and no SWC group (5.80 ± 0.47% vs 5.80 ± 0.82%, *p* = 0.757). None of the models revealed an association between HbA1c or preoperative glycemia and incidence (*p* = 0.794 and *p* = 0.972, respectively) nor grade (*p* = 0.875 and *p* = 0.950, respectively) of SWC.

### Interaction models

**Fig. 3:**
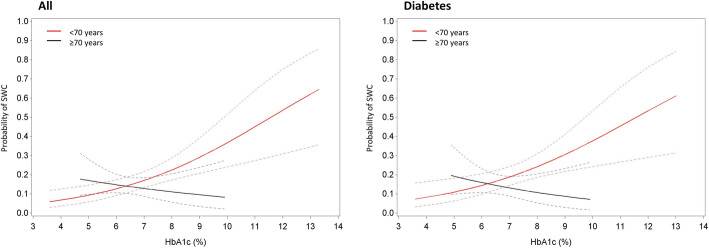
Estimated probability with 95% confidence interval for significant modifiers of the association of HbA1c with sternal wound complications. Estimated probabilities are stratified by age group (< 70 years old versus ≥70 years old) for the full study population (left) and the diabetes population (right)

Variables that significantly modified the association of HbA1c and glycemia with the incidence of SWC are listed in Table 6. In the full study cohort, there was a significant interaction of age group with HbA1c (*p* = 0.021); higher HbA1c was significantly associated with a higher incidence of SWC (OR 1.41, 95% CI 1.17;1.71, *p* < 0.001) in patients < 70 years old, whereas there was no association in patients ≥70 years old (Fig. 3). A similar interaction was seen within the subgroup of diabetes (*p =* 0.021). In the full study population, there was also a significant interaction of urgent surgery with HbA1c (*p* = 0.049); higher HbA1c was significantly associated with a higher incidence of SWC (OR 2.48, 95% CI 1.26;4.88, *p* = 0.009) if the surgery was not urgent, whereas it was not if the surgery was urgent (*p* = 0.914).

**Table 6:** Significant modifiers of the association of hemoglobin A1c and glycemia with incidence of sternal wound complications

Population	Variable	Interaction with	***P*** (interaction)	Stratification	Odds ratio (effect of HbA1c/glycemia)	P (effect of HbA1c/glycemia)
All	Age group	HbA1c	0.021	< 70 years	1.41 (1.17;1.71)	< 0.001
				≥ 70 years	0.85 (0.57;1.25)	0.405
Diabetes	Age group	HbA1c	0.021	< 70 years	1.38 (1.11;1.70)	0.003
				≥ 70 years	0.80 (0.52;1.23)	0.306
All	Urgent surgery	Glycemia	0.049	Urgent	1.03 (0.59;1.80)	0.914
				Not urgent	2.48 (1.26;4.88)	0.009
Diabetes	Skeletonisation	Glycemia	0.029	Skeletonised	4.83 (1.28;18.17)	0.020
				Not skeletonised	0.92 (0.47;1.80)	0.802
Diabetes	BMI	Glycemia	0.029	BMI ≥ 30	0.54 (0.20;1.46)	0.223
				BMI < 30	2.19 (1.01;4.76)	0.047

*BMI* Body mass index

*P* < 0.05 was considered significant

**Fig. 4:**
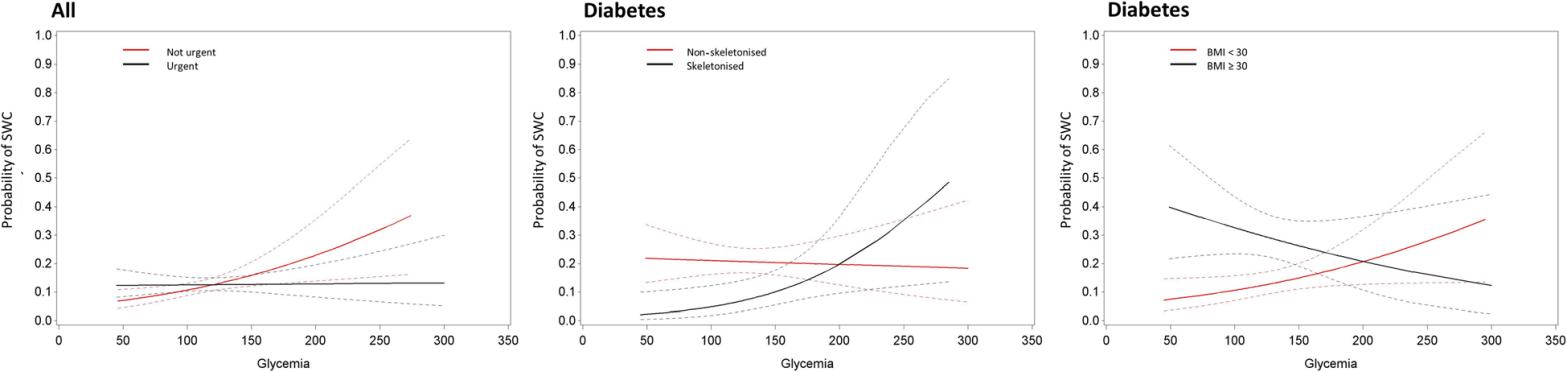
Estimated probability with 95% confidence interval for significant modifiers of the association of preoperative glycemia with sternal wound complications. Estimated probabilities are stratified by urgency (left; urgent versus not urgent), skeletonisation (middle; skeletonised versus not skeletonised), and diabetes (right; BMI < 30 versus BMI ≥ 30) for the full study population (left) and the diabetes population (middle and right)

Within the subgroup of diabetes, a significant interaction of skeletonisation with preoperative glycemia was observed; higher glycemia was significantly associated with a higher incidence of SWC (OR 4.83, 95% CI 1.28;18.17, *p* = 0.020) if skeletonisation was used, whereas no association was present if no skeletonisation was used (*p* = 0.802) (Fig. 4). There was also a significant interaction of BMI with preoperative glycemia; higher glycemia was significantly associated with a higher incidence of SWC (OR 2.19, 95% CI 1.01;4.76, *p* = 0.047) if BMI was < 30, whereas no association was present if BMI was ≥30 (*p* = 0.223).

We found no significant interactions with gender, oral corticosteroids, transplantation, COPD, or BIMA.

## Discussion

### Main findings

This study, investigating 1774 consecutive patients who underwent OPCAB surgery between January 2010 and November 2016, showed that elevated HbA1c was significantly associated with a higher incidence and grade of SWC following OPCAB, although only in diabetes patients and patients younger than 70 years old. An association of preoperative glycemia was found only in patients who underwent non-urgent surgery, diabetes patients who received skeletonised grafts, and diabetes patients with a BMI < 30.

Patients who developed SWC were more likely to have diabetes, a higher BMI, to be of female gender, and more likely to receive BIMA grafts. Even after adjusting for these demographic differences, an increase in HbA1c remained significantly associated with an increased risk of SWC. Of note, diabetes patients who developed SWC were less likely to have received skeletonised grafts, which we have previously shown to be protective against SWC in this population [13].

Although the association between HbA1c and SWC was quite clear in the full study population and the subgroup of diabetes patients, the same relationship was not observed for non-diabetes patients. This finding is in accordance with other studies [14, 15]. Sato et al. [16] provided a potential explanation for this phenomenon by showing that there is a negative correlation of HbA1c with intraoperative insulin sensitivity in diabetes pateints but not in non-diabetes patients. Intraoperative insulin resistance in its turn was associated with an increased risk of infections after surgery. It has therefore been suggested that impaired glucose tolerance during surgery is the actual cause of perioperative hyperglycemia and subsequent SWC, and that HbA1c is an adequate substrate biomarker in diabetes patients but not in non-diabetes patients [17].

Another factor that might in part explain the inability of the present study to find a significant association in the non-diabetes group is that HbA1 measurements were obtained in only a small subset of this group, as HbA1c is only reimbursed in Belgium for patients with known diagnosis of diabetes. The obtained values might therefore be biased because of the reason why they were ordered, which often is the clinical suspicion of diabetes mellitus. Indeed, 68 out of 129 (52.7%) of these patients had a HbA1c between 5.7 and 6.4%, suggesting that they had prediabetes [12]. A HbA1c higher than 6.5% was seen in 8 patients (6.2%), indicating undiagnosed diabetes. Although this sample size was too low to perform separate analyses, a study by Lauruschkat et al. [18] previously showed that patients with undiagnosed diabetes had substantially higher morbidity and mortality rate. This was confirmed in a study of the National Health and Nutrition Examination Surveys (NHANES), showing an increased all-cause mortality in undiagnosed diabetes [19]. The prevalences of undiagnosed diabetes in these studies were 5.2 and 4.7%, respectively, corresponding well with our findings.

Interestingly, we found a significant interaction between age group and the association of HbA1c with SWC: higher HbA1c was significantly associated with a higher incidence of SWC in patients < 70 years old, whereas there was no association in patients ≥70 years old. Because the incidence of SWC was not significantly different between the two groups (17.2% versus 14.0% for < 70 and ≥ 70 years, respectively, *p* = 0.288), this most likely does not reflect an actual lower risk of SWC, but rather an inability of HbA1c to predict SWC in this elderly population. This might be because other risk factors for SWC, other than HbA1c, such as altered immune function, angiopathy, obesity, limited mobility, and malnutrition, are relatively more important in elderly, or because of the impaired basal glucose control with normal aging [20]. Nonetheless, our findings do not necessarily contradict the importance of intensive perioperative glucose control. In fact, HbA1c levels > 8.0% have been associated with increased risk of all-cause and cardiovascular mortality in older adults, highlighting the need for individualized glycemic targets at old age [19].

As glycemia is more sensitive to temporal changes than HbA1c, it was expected that it would contain less predictive power for postoperative SWC. This was confirmed by our finding that in the overall study group, none of our models revealed an association between preoperative glycemia and incidence nor grade of SWC. However, after studying interaction terms, we were able to demonstrate that glycemia did correlate with SWC under certain conditions. Firstly, glycemia was found to be a useful predictor in a non-urgent setting. The stress associated with urgent interventions is known to cause transient disruption of blood glucose profiles, which might blunt their prognostic information [21]. In the setting of urgent surgery, HbA1c might therefore still be a better marker as it is more stable and more readily reflects chronic blood glucose levels. Secondly, glycemia was significantly associated with SWC in diabetes patients with skeletonized grafts or who had a BMI < 30. As demonstrated previously, skeletonisation exerts a protective effect against SWC following OPCAB; thus reducing other risk factors, the effect of dysregulated glycemia might be made more apparent [13]. Similarly, the convergence of multiple risk factors in obese (BMI > 30) patients with diabetes might lead to a blunting of the effect of glycemia on SWC.

### Limitations

This study has some limitations. This was a retrospective observational study and might therefore be susceptible to confounding. However, we identified patient characteristics and used multivariable models to account for characteristics that were different between groups. Nevertheless, this does not exclude that there may have been unidentified confounders that we could not ajust for. As suggested above, only a small amount of the non-diabetes population had available HbA1c values, which might furthermore be biased by the reason for ordering this lab test. A prospective study might therefore be better suited to specifically investigate the role of preoperative HbA1c screening in a non-diabetes population. Finally, we only obtained preoperative laboratory data, and thus could not evaluate intraoperative or postoperative changes in glucose homeostasis.

## Conclusions

In general, elevated HbA1c is significantly associated with a higher incidence and grade of SWC following OPCAB in diabetes patients, whereas glycemia is not. However, the association of HbA1c with SWC seems to be negligible in patients older than 70 years. Furthermore, glycemia might be a useful predictor of SWC under certain conditions: in patients who undergo non-urgent surgery, in diabetes patients who receive skeletonised grafts, and in diabetes patients with a BMI < 30.

## Data Availability

The datasets used and analysed during the current study are available from the corresponding author on reasonable request.

## Abbreviations

BIMA: Bilateral internal mammary artery
BMI: Body mass index
CABG: Coronary artery bypass graft
COPD: Chronic obstructive pulmonary disease
DM: Diabetes mellitus
HbA1c: Hemoglobin A1c
IMA: Internal mammary artery
OPCAB: Off-pump coronary artery bypass
SIMA: Single internal mammary artery
SWC: Sternal wound complication
TX: Transplantion
